# Interpreting Intercepted Communication: A *Sui Generis* Translational Activity

**DOI:** 10.1007/s11196-021-09876-0

**Published:** 2021-12-04

**Authors:** Nadja Capus, Ivana Havelka

**Affiliations:** Université de Neuchâtel, Neuchâtel, Switzerland

**Keywords:** Interpreting, Wiretapping, Intercept interpreting, Court interpreting, Police interpreting, Translation, Criminal law procedure, Multimodality

## Abstract

Legal wiretapping has gained importance in law enforcement along with the development of information and communication technology. Understanding the language of intercepted persons is essential for the success of a police investigation. Hence, intercept interpreters, as we suggest calling them in this article, are hired. Little is known about this specific work at the interface between language and law. With this article, we desire to contribute to closing this gap by focussing particularly on the translational activity. Our study identifies a fragmented field of research due to the difficulty in accessing workers in this specific field who interpret in a highly confidential phase of criminal investigations. The findings, which are drawn from scarce studies and our empirical data derived from an online questionnaire for a pilot study in Switzerland, demonstrate the wide range of the performed activity intercept interpreting. This article is the first to present translational activity from the perspective of intercept interpreters. The activity differs in many ways from interpretation in court hearings or police interviews. Hence, we suggest categorising interlingual intercept interpretation as a translational activity *sui generis* and*—*since previous research has not done justice to the ethical and deontological questions that intercept interpretation raises—advocate for further transdisciplinary research in this field of translation.

## Introduction

Intercepting communication has been a practice ever since the first telegraph poles were erected in 1840 [45, p. 7], and its importance has increased in law enforcement along with the development of modern information and communication technologies. Legal wiretapping to intercept communication is utilised to prevent crime and provide evidence against criminals according to the specific legal rules of each jurisdiction.

An essential factor for successful interception is the ability to understand the content of the intercepted communications. Intercepted persons may speak in foreign languages and, moreover, in colloquialisms, dialects, “regiolects” (a dialect spoken in a particular geographical region), even coded terms specific to their milieu or to obscure a possible police investigation [29, p. 68]. Such linguistic challenges are additionally accompanied by adverse circumstances that hamper the ability to understand the content of the intercepted communications: these adverse circumstances may impair acoustic perception; if the microphone is placed in vehicles or crowded environments, then multiparty conversations may pose difficulties in identifying the speaker.

The actors managing these multimodal challenges are interlingual intercept interpreters, as we suggest naming these participants in the criminal investigation. Their performance is an indispensable pivot in the criminal investigative procedure of fact-finding and evidence construction, and the deployed interlingual translational activity is an important work at the interface between language and law.

Translational activity can be described as a multimodal transfer process that requires a holistic approach to transferring verbal, nonverbal and paralinguistic communication [8; 21; 22; 38; 42]. In legal communication, multimodality plays a major role, it goes beyond verbal and written modalities [28].

Focusing on the modal level, we can observe an intermodal transfer process [21], such as the translation from oral to written text, which is only one of many transfer processes that occur in this specific setting. The diversity and multimodality of source texts found in interlingual intercept interpreting (e.g., live and recorded conversations, text messages) can be examined from the social semiotic point of view embedded in the Translation studies [22; 26].

Further attention needs to be given to the multimodality of “intermedial transfer” [21], e.g., interpreting intercepted communication transmitted by telephone. Different modes and media are involved in the described multiple translation activities in intercept interpreting. Consequently, the aspect "multimodal" implies a dynamic and functional approach to target text production [22, p. 52].

However, scarce knowledge exists about these actors and their activity. With this article, we want to contribute to closing this gap by focussing particularly on the multimodal and interlingual translational activity. First, after briefly presenting methods and material (2) we examine in the following chapter (3) first the terminology (Chapter 3.1) and propose utilising the term *intercept interpreter*, as already mentioned. We then refer to the existing literature and, comparatively, to the literature on police and court interpretation to better highlight the special features of this activity by contrasting it with court and police interpretation, as these legal interpretation services are deployed in the same context: the context of criminal procedures (Chapter 3.2). The scarcity of existing research led us to reflect on its potential reasons (Chapter 3.3). In Chapter 4, we present initial results from the empirical material that we gathered through our pilot study: an online survey of intercept interpreters in Switzerland in 2020–2021. The material reveals valuable information about the actors’ personal and educational profiles as well as about the experience that intercept interpreters personally have with the translational activities they are required to provide. The aim is to explain the translational activity and the profile of intercept interpreters by drawing on empirical data from an online questionnaire.^1^

In this context, we must specify that this article does not focus on one jurisdiction in particular, as we are not discussing legal issues, such as legal regulation of communication interception or evidence law. However, as the empirical material was gathered in Switzerland, we must note that Switzerland is a civil law country with rather inquisitorial criminal procedures. Hence, the police and the public prosecutor’s office initially investigate a case to establish the facts, gather evidence, and collect details in a case file. If correctly gathered, then the work product of intercept interpreters enters the procedure as part of the case file in the form of written records in the language of the procedure.

Finally, we conclude from the findings of Chapters 3 and 4 that intercept interpretation does not fit into pre-established categories and that it should be categorised as a translational activity *sui generis* (Chapter 5). Furthermore, the wide range of translational and forensic activities deployed by intercept interpreters as well as the important differences in the applied translational transfer strategies (as compared to court and police interpretation) indicates that it would be insufficient and even misleading to extract conclusions regarding intercept interpretation from the—until now—better researched area of court and police interpretation. Further transdisciplinary research is needed to enhance knowledge and subsequently allow improvements to the formation, professional training, and practice standards of intercept interpreters.

## Methods and Materials

The online survey was conducted among intercept interpreters working in Switzerland (2020–2021). Reaching this group represents one of the most difficult challenges when conducting empirical research in this field. The difficulty begins with the basic information about the number of intercept interpreters employed in Switzerland. No information is available, and even an estimation is difficult to provide because intercept interpreters are hired on a district level, mandated by cantonal police authorities depending on their secret surveillance activities; consequently, no statistical data are accessible regarding this employment system. One could imagine that annual budgetary reports from the police or public prosecution office would at least offer some indications, but if these reports are accessible at all, then the mentioned costs indicate legal interpretation services without differentiating between court hearing, police interview, and intercept interpretations.

Moreover, authorities do not proffer lists of the mandated intercept interpreters, nor are these professionals organised in associations. Police and public prosecution authorities generally guarantee the confidentiality of intercept interpreters’ identities. Hence, our access was necessarily indirect and would not have been possible without the active support of the responsible police and public prosecution offices, as they forwarded the survey invitation by email to the intercept interpreters in their district. We were not provided information regarding whether all the institutions we contacted forwarded the invitation.

We developed a questionnaire comprising 157 questions that are divided into five sections. The questionnaire was developed in English and translated into German and French. Participants could choose one of the language versions. The estimated duration to answer all questions was approximately one hour; therefore, participants were remunerated CHF 70, which is the standard hourly rate for intercept interpreters in Switzerland. We guaranteed anonymity of the answers. The only participation criterion was that participants must have worked as an intercept interpreter in a criminal case in the past four years.

The data summarised in this paper were collected between December 2020 and July 2021 and include 46 complete sets of responses (of a total of 87); 34 were in German, 10 were in French and two were in English. Of the 46 respondents, 30 were female, 14 were male, and one indicated other and one person did not respond to this question. They ranged in age from 23 to 63, but only five respondents were younger than 35. Professional experience as an interpreter ranged from one month to 28 years, with 14 respondents reporting more than 10 years of experience.

Before discussing our findings (Chapter 4), in the theoretical part (Chapter 3), we explain the presentation of the translational activities of intercept interpreters in the existing literature. Inevitably, the presentation is fragmentary, as only scarce literature is available regarding intercept interpretation. To better highlight the special features of this activity, we draw from two other sources: first, we find it revealing to contrast intercept interpretation with court and police interpretation, as they are legal interpretation services of proximity, as both are deployed in criminal procedures. Second, though focussing on the interlingual translational activity, we include knowledge gained from empirical intralingual translational activity [3; 22; 36], as intralingual intercept interpreters are confronted with similar tasks, particularly the task of deciphering codes. Intralingual translation is considered to be a transfer or rewording within one language, while interlingual translation is the transfer between two languages [33, p. 5].

## Intercept Interpretation: The Academic Perspective

### Terminological Muddle

Seeking an appropriate name for the interlingual actors in communication interception in police investigations allows access to an impressive variety of nomenclature.

Primarily, the terms *interpreting* and *interpreters* are utilised [14; 15; 18; 39]. However, the compound term *interpreter/translator* [18] is utilised as well. Similarly, in German, the terms *Dolmetscherin/Dolmetscher* (interpreter) and *Übersetzerin/Übersetzer* (translator) are both utilised [15]. Therefore, the question arises regarding whether intercept interpretation in fact encompasses both activities: interpreting as the oral rendering and translating as the transfer from written texts [7; 29].

Yet another feature of translational hybridity in intercept interpretation is that it constitutes highly technologically supported forms of translational activity [39, p. 5] based on a variety of media sources (such as oral conversations and short text messages). Hence, distinguishing between translating and interpreting based on written versus oral rendering does not seem to be a sufficient criterion [37, p. 11].

Scholars are attempting to create new designations to emphasise the autonomy of the activity, as distinguished from translation or interpretation. Drugan [9] utilises the term *linguist* to denote a provider of language services that may differ from translating and interpreting. In fact, the term *forensic linguistic expert*, as it has been proposed from scholars in Belgium, emphasises the investigative aspects of the work [39]. However, Belgium Law uses the term “translators-interpreters". In Switzerland, as another example, the Language Services Ordinance of the Canton of Zurich, which was enacted on 1 July 2019, refers to linguistic mediation (‘Sprachmittlung’) in the field of communication interception [5, p. 75].

One possible criterion may be the presentation and availability of the source text. Otto Kade argues that the deciding factor is not an oral or even motor-phonetic or acoustic [19, p. 34] mode of presentation; rather, the determination is based on its availability and repeatability and thus also the question of controllability of the rendition. Kade’s definition proposes that interpretation is performed under time pressure and with only minor corrections, while *translating* is defined as a translational action with a static source text. The source text and the source medium can impact the translation regarding the translational strategies utilised. Interpreting in a live, intercepted communicative event requires different translation strategies than translating a written text, such as a transcript of a conversation.

In sum, the two conventional categories (interpreting and translating) as such fall far short of capturing the full range of activities and resulting hybridity, as the next subsection reveals. Hence, our proposition to utilise the term *intercept interpreters* is a compromise: it does not break altogether with the conventional terms but clearly indicates the special circumstance of the multimodal and intermedial interpretation activity. However, it has the disadvantage of not clearly highlighting the whole scope of the activity.

### The Hybridity of Translational Activities

The previously established variability regarding the designation of translational agents in intercept interpretation may be an indicator of the hybridity of translational activities.

The purpose of intercepting communications—which is defined as the legal wiretapping of landline or mobile telephone communications and bugging of cars, apartments, or any other location—is generally stated to be the collection of information and evidence during police investigations [3; 9; 14; 18; 36; 39]; however, the concrete contributions of intercept interpreters is less clearly identified.

In wiretap operations, the linguistic transmission, both interlingual and intralingual, occurs unidirectionally and typically under time pressure. In fact, as intercepted persons are not informed about the wiretapping during their communicative event, their communicative behaviour is therefore not influenced by the interception.

Intercept interpreters are required to identify and contextualise auditory content, sometimes within seconds [14, p. 114]. The high degree of spontaneity in communicative events and interlocutors combined with emotionally stressful conversational content is one of the particular difficulties of this type of work. In addition to rapid, slurred, or imprecise speech, interruptions and overlapping auditory segments (several people speaking simultaneously), the usage of regionalisms, slang or in-group language specific to particular communities, and the usage of coded language as well as code switching is prevalent. This situation differs completely from legal interpretation in court or police settings, which is usually deployed consecutively and bidirectionally [2; 7; 10; 16; 20; 29]. Modes of interpretation utilised in this area include chuchotage (whispering), sight translation [2; 7; 10; 20; 29], and summary interpretation [2; 10; 13; 20; 29]. In fact, dyadic communicative events form the usual interpretation situation in communicative settings at court hearings and in police interviews. These include at least two primary interlocutors, with the interpretation being bidirectional and primarily dialogic, to provide immediate exchange and understanding.

Furthermore, the institutional embeddedness in court hearing and police interview settings differs from the intercept interpretation setting: in court and police settings, the communicative action is coordinated top-down in the sense that judges, prosecutors or police officers control the interview situation, ensuring that the interpreter has sufficient time at their disposal and the ability to clarify questions of comprehension. This also ensures that persons who are deposing their statement via an interpreter may undertake corrective actions if they recognise a misunderstanding or other error [25]. The communicative action transpires as a planned and highly standardised form of communication [40, p. 113], in which all actors are assigned fixed tasks. Contrary to this kind of communicative action, interpreting intercepted communication is dictated by the spontaneous communicative behaviour of the intercepted persons outside an institutional context. Questions clarifying the intended meaning cannot be posed. Intercepted persons have no influence on the selected and interpreted content resulting from the interception nor are they able to take immediate corrective actions. This contributes to the general problem that the work of intercept interpreters is difficult to verify regarding the correctness of content [17, p. 26].

According to some researchers, special attention is needed regarding the technological features of the intercept interpretation setting. The activity is conducted remotely without visual cues and is mediated by technology; consequently, digital competences are required to produce high-quality target texts [14, p. 114]. Hence, Gradinčević-Savić [15, p. 182] explicitly mentions IT skills as a requirement for intercept interpreters, as they must be able to work independently utilising IT interception systems.

Moreover, the communicative action is reconstructed based on auditory cues when oral communications are being intercepted. Accordingly, González Rodríguez [14, p. 114] sees a strong connection between the requirements for interception interpretation and telephone interpretation (*interpretación en/para escuchas telefónicas*): both rely on mono-sensory perception via the auditory channel. Recognising and assigning voices is paramount for intercepting communication via audio [3; 9; 14; 36]. During an operation, the interpreter may be required to recognise several voices, some of which may overlap in conversation. However, the voice as a multimodal source of auditory information reveals more than just the identity of the speaker; it provides verbal, paraverbal (tone, pacing, volume), and nonverbal communication (e.g., socially relevant information as style and tone) [11]. The specific cultural group to which speakers belong, their level of education, and other social characteristics can be determined at the verbal level. At the paraverbal or nonverbal level, information can be extracted regarding age, gender, personality, ethnicity (e.g., based on the accent) as well as health or the emotional state [11, 98ff]. Finally, through ambient noises, kinesics allows the listener to draw conclusions about the speaker’s location and the environment in which the conversation occurs [14, p. 117]. Acoustic obstacles may impede understanding of the communication; these can result from background noise, the speaker’s pace of speech, pronunciation that features a strong dialect or accent, several persons speaking simultaneously, or incomplete sentence structure. Also, the listener’s knowledge and expectations can have an impact on speech perception [24].

An additional distinction from court hearing and police interview interpretations is that the main purpose of translational activity in interception settings is not to facilitate the communicative goal of the primary interlocutors. Rather, the core activity is working closely with police officers to collect information and produce evidence in the form of translated transcripts of intercepted communication for usage in court proceedings [9; 15; 17; 18; 39]. Interception work requires strict co-operation between investigators and intercept interpreters [9, p. 318]. Throughout the interception process, interpreters remain in continuous exchange with the investigative team [14]. However, the exact level of relationship between intercept interpreters and police investigators as well as public attorneys is unclear in current research.

Research strongly indicates that intercept interpreters are expected to have a high level of investigative competence, as their main task is to gather information and evidence [9, 313f; 36, p. 28]. Depending on the investigational relevance of the intercepted content, these results may be utilised in preliminary court proceedings. Extant research does not indicate that intercept interpreters are trained similarly to investigators; rather, it seems each interpreter develops their own investigative intuition [17, p. 25]. Intercept interpreters are expected to detect allusions, subtle nuances, or ambiguities [17, p. 25]. Filtering potentially relevant from irrelevant content while overhearing the conversation almost simultaneously constitutes a stressful task [9, p. 313; 36, p. 30].

Research reveals that this activity is diverse, multilayered, and stressful. The diversity of translational activities for intercept interpreters is determined by the various types of source texts: auditory information in real time, recorded conversation segments, or short written messages. Moreover, language codes must be recognised and decoded, and relevant content must be sifted from large amounts of irrelevant conversational material [9, p. 313]. Hence, a central activity consists of extracting information that incriminates the intercepted person, which leads to ‘entextualisation’: the process of cutting sections of conversations out of longer sequences [3; 36; 41; 43].

Intercept interpretation is not oral and therefore transient; rather, the goal is to produce a written record to be utilised in further proceedings. Whether a verbatim or a summarised translation is required [17, p. 25] seems to depend, at least in Switzerland, on circumstantial factors and the appreciation of the police and prosecutorial authorities. However, the respective instructions provided to the intercept interpreter should be documented in the case file [5].

Transcripts are an important work instrument because they provide a time-saving overview of the audio recordings that are collected throughout the proceedings [13, 965ff]. In a twofold process, the intercepted communication is transferred from its spoken form into written summaries or translations and reorganised into institutionalised categories with legal relevance, which results in its decoupling from the original communicative event [3, p. 505; 36, p. 31]. Consequently, the transcribed utterances are regarded more as an instrument and less as a documentation of the spoken communication [36, p. 28]. Providing an impartial approach is considerably difficult, since commissioning a transcript or translation serves an explicit purpose: to incriminate one or more interlocutors overheard in the recorded communication. Therefore, transcripts and translations are commissioned based not only on the intercepted utterances but also on the current knowledge of the investigative team [3, p. 509]. Thus, the findings of previous investigations influence the translational activity, especially regarding the language decoding that is expected of interpreters [9, p. 314]. Transcripts are produced largely under the supervision of the author (also known as the auditor, interpreter, or transcriber) and the institution for which they are authored [3, p. 505]. Spoken content is reviewed for relevance to the investigation, and code words and nicknames are interpreted semantically [36, p. 34]. It remains unclear from the existing studies whether and how transparency is assured, that is, whether and how any ambiguities in the target language can be traced to the original utterance.

Hence, translational activity of intercept interpretation is a hybrid activity which differs in myriad ways from that required for interpretation in courtrooms or police interrogations; therefore, in our opinion, it should be categorised as a translational activity *sui generis* in the legal context.

### The Lack of Research on Intercept Interpretation: The Difficult Accessibility of the Research Area

In a previously conducted literature review on intercept interpretation [6] we showed that abundant literature exists regarding legal interpretation in criminal procedures (court hearing and police interview interpretations), but literature is scarce regarding intercept interpretation. This finding is statistically confirmed by the bibliometric analysis from Monteoliva-Garcia [30]. She identifies over 400 studies (*n* = 464) on interpretation in court and for public authorities; her analysis covers the period from 2008 to 2017. These studies include interpretation in all phases of criminal proceedings (in court, with the police, and in prisons), in asylum and immigration systems, and in the military and for other jurisprudential communicative events. A quantitative analysis reveals a clear focus of scientific interest in court interpretation (54%) [30, p. 46], and a growing interest in police interception (see, for example, the *ImPLI—Improving Police and Legal Interpreting Campaign* at the European level^2^ or Transnational Organised Crime and Translation [TOCAT] [44]), while intercept interpretation studies are lacking.

One could assume that this dearth is simply because fewer intercept interpreters than police and court interpreters work for criminal justice systems. However, more substantive reasons explain the lack of research. Researchers’ focus on interpretation in court settings may provide a clue regarding the main reason for the lack of research on intercept interpretation: the feasibility of empirical research strongly depends on the accessibility of the research setting. Accordingly, court hearings have the distinct advantage of being subject to public disclosure, which is an important pillar of any constitutional state, ensuring that all court hearings must, in principle, be open to the public. The principle is anchored not only in national legal systems but also in international conventions, such as the European Convention on Human Rights (Article 6, Paragraph 1) or the International Covenant on Civil and Political Rights (Article 14, Paragraph 1, Sentence 2). This means that court interpretation is more accessible, transparent, and easily observed by interested researchers [1, p. 107] than the work of interpreters engaged in wiretapping activities for the police force.

To date, professional access seems to have been essential to opening doors for research on intercept interpretation [3; 9; 14; 15]. Consequently, empirical studies have been provided by practicing interpreters, which makes them so-called ‘practisearchers’. This kind of research approach is characteristic for police interpretation research in general: notably, with few exceptions, such ‘practisearchers’ have delivered pioneering work in this area [2; 12; 27; 31; 32; 34; 35; 40; 46].

Furthermore, the scarcity of research may represent a negative circular flow: lack of accessibility leads to less research, less research leads to non-existent knowledge and lack of awareness regarding the inherent risks of intercept interpretation, which in turn hinders criminal law authorities from welcoming research and providing access. To complement the research approach toward intercept interpretation, we present in the next chapter preliminary results from a pilot study that utilised an online survey with intercept interpreters.

## Intercept Interpreters: Who they are and How they Perform Translational Action

### Findings from the survey

As existing literature is silent on the professional background of intercept interpreters, the questionnaire includes questions about this issue. Notably, only a small proportion of the intercept interpreters surveyed in this Swiss case study (*n* = 9, 19.57%) are members of a professional association (Question 20, question is hereinafter abbreviated as Q). The educational level ranges from PhDs (*n* = 2, 4%) to secondary school diplomas (*n* = 2, 4%); some of the respondents had earned vocational certificates (*n* = 8, 17%; Q143). Slightly less than one-fourth of the respondents indicates having a bachelor’s degree as their highest qualification (*n* = 10, 22%). The same number of respondents indicated having a master’s degree as their highest qualification (*n* = 10, 22%). However, of all respondents, only two state that they have a bachelor’s degree in translation (Q145). The other degrees are from various disciplines, such as law, modern languages, business administration, and communication sciences. Regarding vocational trainings, the majority of respondents reports that they have attended various introductory courses or in-house trainings offered through the courts or professional associations on translation and/or interpreting (Q151). Nevertheless, 76.09% (*n* = 35) of all respondents consider themselves to be translators or interpreters, 17.39% (*n* = 8) refer to themselves as bilinguals without relevant training, and three respondents identify themselves as teachers (6.52%; Q156).

The requirements are demanding and diverse regarding language and cultural knowledge, translational skills, and technical handling, as reported in Chapter 3.2; however, we observe that in Switzerland no specific formation seems to be required for this job, and the necessary skills are provided through on-the-job training. Even the recruitment of new intercept interpreters is not conducted through an official job vacancy announcement that notes required specific certificates and skills; rather, hiring often materialises on a personal level through personal networks (Q21, 25). Consequently, training in the setting varies significantly depending on the police station. Slightly more than one-half of the interviewees has received no training or briefing at all before their first deployment, but only an explanation of the technical system and software, whereas the other one-half attended courses and received detailed training (Q33).

Responses seem to indicate that intercept interpreters may develop a certain routine if they are assigned to long-term investigations or several investigations concurrently or successively. However, intercept interpreters state that they may be mandated for a few hours or for a few years (Q51). Slightly more than two-thirds of the respondents are sometimes involved in two to three different investigative acts in parallel. Only a few respondents report that they work in as many as five investigative acts at the same time (Q53). Almost all respondents state that they are occasionally called for an emergency (Q65). Two-thirds of all respondents report that they also interpret (*n* = 31, 67.4%) at night, between 11 pm and 7 am (Q68).

The interpreters’ lack of a professional organisation or membership in professional associations may be related to the nature of the activity, which is confidential and hidden from public view to protect not only the success of the investigation but also the intercept interpreters involved. Hence, discretion is understood as an essential aspect by the respondents. This might have been the inner attitude of respondents when answering the questionnaire. It seems, indeed, that the discretion and confidentiality which accompany the activity itself, render intercept interpreters themselves almost invisible in many facets [5]. Intercept interpreters in Switzerland are well aware that they are bound to secrecy regarding facts that come to their knowledge in the course of their translation work (Q100). A breach of this official secrecy may be punished by a fine or imprisonment. Furthermore, confidentiality is in intercept interpreters’ interests so that their identity and involvement with the interception is not revealed. This primarily protects the intercept interpreters and their family members from retaliation attempts. The following quote illustrates the impact of the work on the personal environment of intercept interpreters. When asked about difficulties associated with the job, one respondent states (we translated this answer to Q110),

Threats, insults from the accused. Our role as interpreters is often misinterpreted by our compatriots and the accused. We are sometimes called spies by our compatriots. You have to live very carefully and quite reservedly.^3^

Confidentiality is in fact one of the obligations mentioned in response to queries regarding intercept interpreters’ three most important obligations. The other two predominantly mentioned obligations are complete and truthful translation as well as impartiality. However, as we explained in Chapter 2.2, performing an impartial approach is considerably difficult, since commissioning a transcript or translation serves an explicit purpose: to incriminate one or more interlocutors overheard in the recorded communication; furthermore, intercept interpretation transcripts are produced largely under the supervision of the police officer who is in charge leading the interception and represents the institution for which they are authored [3, p. 505]. Hence, we were interested to learn more about the specific tasks and instructions that intercept interpreters receive from the police. Participants indicate that they are instructed regarding how to proceed with translational strategies, such as how and what to translate (Q34). Notably, the goals of intercepted communication are explained, and the intercept interpreters are provided with information such as the facts of the case, the relevant criminal offences, the language or dialect, and the description of the target person (Q50). Additionally, intercept interpreters were asked to indicate what types of translations of intercepted communications they perform. Multiple answers were possible. Most of the respondents answered to be performing a verbatim 1:1 transcription of the whole communication but they stated also to be performing summaries of the communication. Also, notes (comments of the intercept interpreters in translations) indicating trivial or irrelevant content of communication are submitted (Q76).

To undertake this highly selective task, intercept interpreters are instructed by police officers regarding which content requires special attention (Q77). This can include information on the hierarchy of a perpetrator group, the contacts and relationships among them, or their place of residence and movement behaviour.

However, the manner in which police officers in Switzerland offer instructions seems to be highly individual: a common standard is lacking. Indeed, when asked how they decide which content to translate, the majority of respondents states that they base this decision on the information provided by the police officers on the one hand but also on their own case knowledge and relevance to the criminal proceedings on the other hand. Significantly, one respondent mentions having personal *‘common sense and a criminal streak’* are helpful characteristics (Q75). The following quote illustrates that intercept interpreters have an investigative role as well (answer to Q77):

It depends on the subject. By listening to the communication, I notice if the information is important for the investigation or not. If it is important, I transcribe it word by word; if it is about trivialities, a short summary is enough.^4^

One respondent explains that the preferable approach is as follows (answer to Q107):

Depending on the conversation, pay attention to the elements related to the offence only (e.g., if it is about narcotics offences, then I will pay more attention to details for narcotics and less for burglary). The same goes for human trafficking and money laundering (more attention for these offences and less for, for example, […] burglary).^5^

Hence, on the one hand, the rendering strategy is determined by police officers, but on the other hand, intercept interpreters are also entitled—as they indicated—to decide based on ‘*experience*’, ‘*according to relevance*’ or due to ‘*case knowledge*’. Regarding relevance, one respondent clarifies to make decisions ‘*according to the importance of the conversation, its content, the importance of the person in the investigation*’ (Q77). Regarding the expectations of the police officers, the participants provide varying responses. The following quote illustrates the challenge of meeting different expectations (answer to Q109):

For some, it’s a rush, for others, less so. Some only want the most important things written down; others also want background knowledge that at first glance is not relevant to the crime to be written down, etc.^6^

**Fig. 1:**
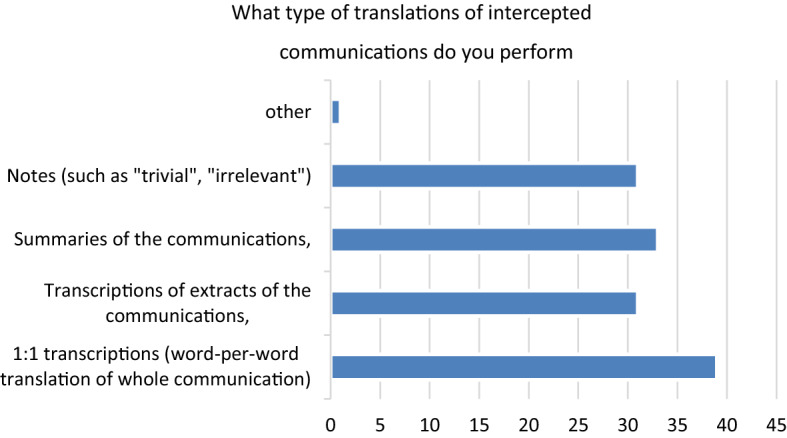
Q76 What type of translations of intercepted communications do you perform?

As for the concrete product, the variety continues: intercept interpreters are either asked to provide a transcript in the original language, a summary, or an excerpted or complete verbatim translation of the oral material (Fig. 1). When excerpt translation and decoding are requested, then intercept interpreters indicate that the police officers decide on the procedure (Q79). But only the way transcripts are to be provided differs. The situation of the interpretation itself may also differ profoundly: intercept interpreters are either asked to provide live interpretation or subsequent interpretation based on pre-recorded interceptions.

**Fig. 2:**
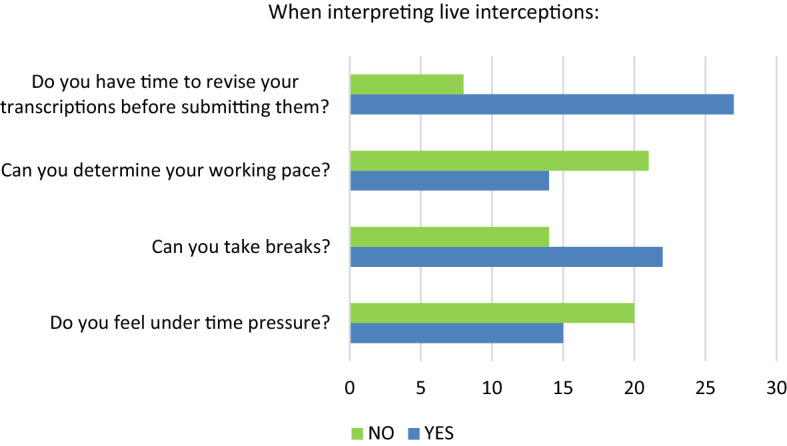
Question 80, #1—When interpreting live interceptions

According to intercept interpreters, submitting a written translation from a live interpretation is delivered under slightly more time pressure and demands more resilience and flexibility since the working pace and work load are unknown (Q80; see Fig. 2).

**Fig. 3:**
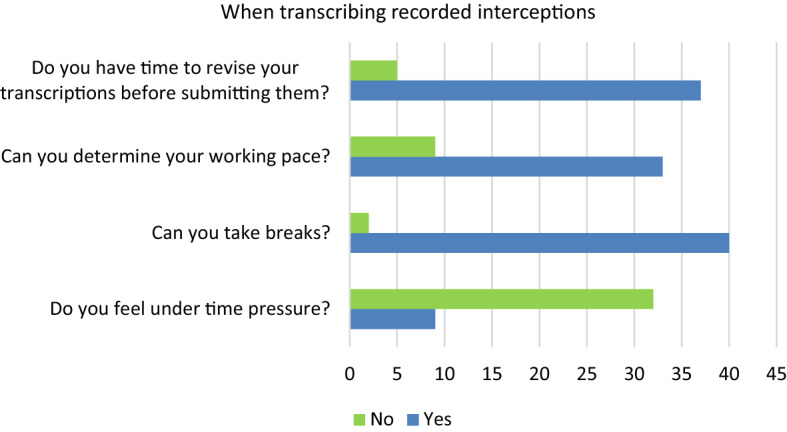
Question 81 #1—When transcribing recorded interceptions

Conversely, transcription and translation of pre-recorded interceptions is considered to be to some extend less stressful because breaks can be taken and more time is allowed for correcting the written translation (Q81, see Fig. 3).

Based on the information from the survey, different procedures seem to be utilised within the Swiss cantons and police stations regarding whether a transcript is produced in the original language before translation (Q77).

Police stations are the workplace for intercept interpreters. Notably, almost all intercept interpreters interviewed (*n* = 43, 93.5%) indicate that they work at the police station and closely with police officers who are directly involved in the case (Q101). Often, they work with several police officers concurrently (Q102). Technical equipment and office space exclusively for interpretation and translation are provided (Q89), but one essential tool is especially desirable for the activity: an Internet connection (Q85). We assume that this is utilised in particular to check city names, geographical distances, or even terminology. Additionally, other intercept interpreters and police officers are present in the immediate work environment (Q93). This helps accelerating the communication: intercept interpreters indicate to receive feedback for the translations, such as figures or details on times; this is usually provided orally (Q96). In addition to contact with police officers, intercept interpreters are in contact with the public prosecutor’s office; for example, they may be asked to provide ad hoc interpreter services during an interrogation or to provide written translations (Q123).

However, the performed activity is not restricted to the translational activity. Two-thirds of all respondents (*n* = 31, 67.39%) state that they are also asked for their opinion regarding a wiretapped situation. Specifically, they are asked about their personal assessment of a situation or person. In addition to cultural backgrounds, linguistic nuances, such as pronunciation and coded language, as well as the mood of the intercepted persons must be assessed (Q115).

Regarding the control of their services as intercept interpreters, only a few respondents (*n* = 8, 17.39%) experienced a subsequent challenge to their translations. Such objections were raised by defendants or their defence counsel at trial (Q126).

## Discussion

The method in which the pilot study was conducted demonstrates the extent to which intercept interpreters are a hard-to-reach professional group. This may be due, on the one hand, to their highly assimilated obligation to maintain confidentiality. On the other hand, intercept interpreters are a heterogeneous group, which is greatly reflected in their educational levels and personal backgrounds, and this may also have impacted our difficulty in finding them. Regarding their professional backgrounds, one important finding of the pilot study is that although the majority of the participants considered themselves to be professional interpreters, the activity as such seems to be the only common denominator. They were not organised within a professional association and have not typically applied for membership in any professional association; they did not share common educational formation apart from introductory courses or in-house trainings. Furthermore, the initial findings of our empirical data indicate that intercept interpretation is often conducted by untrained and natural interpreters whose only common denominator is bilingualism—a fact that is accepted in Switzerland, where the survey was conducted, by involved police and public prosecution offices that mandate intercept interpreters as well as by courts as high as the Federal Supreme Court [5].

Our findings based on this Swiss case study confirm that intercept interpreters perform on multiple levels due to the complexity of the task; however, they also reveal the obscurity surrounding the scope of translational activity. Additionally, the technique and the source material utilised (live conversations, recorded conversations, and transcripts) influence the translational results and therefore must be indicated and considered when reading written reports from intercept interpreters. However, it seems that such decisions or translational strategies change from case to case and that no clear criteria are being followed. Translational strategies may even change within one intercept interpretation mandate. Intercept interpreters indicated that translational transfer strategies are not specified and depend on the police supervisor. Furthermore, the answers regarding translational transfer strategies demonstrate that a standard approach is lacking. The appropriate procedure remains obscure. Even decoding, a rather delicate task, is, in the Swiss criminal justice system, acceptable to perform in an informal setting. Formally, the Federal Supreme Court upholds the exclusive authority of the police and the public prosecutor to interpret coded language and implicatures (utterances implicating something even though not literally expressed). This renders the intercept interpreter invisible and places the investigators in the foreground, while the intercept interpreter’s decisive contributions to successful investigations remains in the background [5]. This disturbing reality collides with the requirement to render the mode of evidence production transparent [5, 345ff].

The labour division and co-operation between police investigators and intercept interpreters remains deeply obscure. From a purely contractual perspective, the majority of intercept interpreters are independent professionals whose efforts are mandated for concrete cases. From a legal perspective, they are considered to be neutral experts [5]. However, intercept interpreters seem to perceive themselves as members of the investigative team, although they perform investigative activities without receiving specific training.

## Conclusion: A *Sui Generis* Activity with Important Elements That Remain Obscure

Knowledge regarding the paramount work of intercept interpreters at the interface between language and law is fragmented and must be further developed. To close this gap, we have examined the terminology employed. Literature regarding interpretation studies reveals inconsistencies in the designation of translational agents in intercept interpretation. However, these inconsistencies are due to the variety of tasks that intercept interpreters are asked to perform. Accordingly, research has revealed the extent to which intercept interpretation covers a wide range. Our analysis of the existing literature, contrasting the findings with research on court and police interpretation, indicates that hybridity, variety, and diversity regarding translational transfer strategies do not allow us to draw any conclusions from studies on court and police interpretation.

Accessibility is the predominant issue when researching this topic empirically. Therefore ‘practisearchers’ have delivered pioneering work in this area. Indeed, a circular effect transpires: intercept interpretation is performed during a delicate phase of police operations as secret investigations are ongoing. Thus, confidentiality must remain the priority. Furthermore, law enforcement authorities have no interest in disclosing their working strategies and tactics in this field to the public. The resulting lack of research does not raise awareness of the problems related to the language work occurring in this stage of criminal procedures. Hence, to raise consciousness and awareness, which in turn will render the field more accessible, other approaches should be developed.

We attempt to contribute to this dearth of knowledge with our online survey. The findings from the study confirm and complement important issues raised by existing literature: intercept interpretation consists of highly delicate activities that extend far beyond the strict translational action of transferring spoken and written language to include forensic linguistic activities. Nevertheless, insufficient linguistic, translational, and technical skills of intercept interpreters represent important risks for efficient and fair criminal investigations. Furthermore, no special formation is required, and only on-the-job training is provided. Intercept interpreters are typically untrained persons whose only common characteristic is bilingualism.

Moreover, our findings reveal that a standard process is lacking in the recruitment, formation, and operational work [4; 5; 23]. This result is corroborated by literature that refers to other states, such as Germany [15] and Belgium [39]. Developments in other fields, such as court and police interpreting, indicate that awareness only increases after empirical findings become available. Improvement of practice by establishing minimal standards regarding formation and professional training as well as instructional practices employed by authorities, such as police and public attorneys, must follow.

While discretion and—in justified special circumstances—anonymity is plausible and comprehensible, obscurity in translational transfer strategies, labour division, and relationships between police officers and intercept interpreters is not. In our view, the established obscurity regarding translational transfer strategies, fuzzy labour division, and unclear relationships between police officers and intercept interpreters raises important ethical and deontological issues that need to be further investigated. Furthermore, the technique applied and the source material utilised (live conversations, recorded conversations, or transcripts) influence the results. Therefore, the lack of transparency in this context is unacceptable. Additionally, intercept interpreters perceive themselves as being part of the investigative team, and they perform investigative activities. However, the attribution of related responsibilities remains obscure, and important tasks, such as decoding, generally occur in an informal setting. Non-transparency regarding translational strategies, techniques applied, and source material utilised as well as the fuzzy distribution of responsibilities between police investigators and intercept interpreters are worrisome, as intercept interpretation material may ultimately become evidence or at least lead to evidence [4, 345ff].

We hope that this article contributes to raising the necessary awareness and to encouraging further empirical and transdisciplinary research. In our research project,^7^ from which the questionnaire issues, we utilised different datasets and methods, such as case file analyses, interviews, participative observation, expert workshops, and case studies that are based on audit record and intercept interpreter transcripts and subsequently provided own translations which allow to compare the different versions. A replication of the questionnaire in different countries would lead to richer data. Based on our experience, however, we suggest reducing the number of questions. Moreover, to complement research revealing the perceptions of intercept interpreters, future research could, for instance, focus on police officers’ perceptions of the role of intercept interpreters and how they perceive this collaboration.

## Footnotes

1 The questionnaire was developed by Dr. Cornelia Griebel, Dr. Damian Rosset and Prof. Nadja Capus as part of the interdisciplinary research project “Intercepting with interpreters” directed by Prof. Nadja Capus and funded by the Swiss National Science Foundation (2019–2022).

2 https://eulita.eu/impli-improving-police-and-legal-interpreting/

3 Own translation from German.

4 Own translation from French.

5 Own translation from French.

6 Own translation from German.

7 Research project “Intercepting with Interpreters”, financed by the Swiss National Research Foundation (project n° 100011_184896), https://www.unine.ch/crrc/intercept-interpret.
